# Anisotropic zero-index waveguide with arbitrary shapes

**DOI:** 10.1038/srep05875

**Published:** 2014-07-29

**Authors:** Jie Luo, Yun Lai

**Affiliations:** 1 College of Physics, Optoelectronics and Energy & Collaborative Innovation Center of Suzhou Nano Science and Technology, Soochow University, Suzhou 215006, China

## Abstract

We design a series of waveguides composed of uniform anisotropic epsilon-near-zero media. Unlike normal waveguides in which the transmission rate strongly depends on the width and the boundary shape, such waveguides can achieve high transmission with almost arbitrary width and boundary shapes, leading to applications such as unusual waveguides, wave expanders and compressors, splitters, bends, and devices with combined purposes. The physical origin of such high transmission can be explained by using transformation optics and the condition for total transmission is derived. Numerical simulations with multilayers consisting of dielectric and negative-permittivity materials proved our theory. Our work provides a unified physical picture for waveguide structures based on anisotropic epsilon-near-zero media.

Novel microwave and optical devices have been proposed based on metamaterials exhibiting almost arbitrary effective permittivities and/or permeabilities, including perfect lens^1^, invisibility cloaks^2^,^3^,^4^ and illusion devices^5^,^6^. As a special kind of metamaterials, epsilon-near-zero (ENZ) and/or mu-near-zero (MNZ) materials exhibiting near-zero permittivity and/or permeability, respectively, have been extensively investigated, and various applications have been proposed, such as directive emission with controllable wave front shapes^7^,^8^,^9^,^10^,^11^, squeezing and tunneling electromagnetic waves in narrow waveguides^12^,^13^,^14^,^15^,^16^, designing optical circuits^17^,^18^, manipulating transmission by engineering defects^19^,^20^,^21^,^22^,^23^,^24^, etc. For waveguides composed of isotropic ENZ or MNZ materials, the area of waveguides are required to be vanishingly small to achieve high transmission^12^,^13^,^14^. However, for waveguides composed of double zero materials with both permittivity and permeability near zero^16^, or anisotropic ENZ or MNZ (AENZ or AMNZ) materials^25^,^26^,^27^,^28^,^29^, high transmission can be achieved with a finite area. Double zero materials are more difficult to achieve than single zero materials. Therefore, AENZ or AMNZ materials are more feasible for applications such as directive emission^7^,^11^ and power combination^9^,^10^. Recently, we have proposed to use AENZ or AMNZ materials to realize a bending waveguide of arbitrary angle and length^25^, which has been experimentally verified^26^. And base on the inhomogeneous AENZ media, we have proposed a way to achieve arbitrary manipulation of electromagnetic energy flux in sub-wavelength scales^29^, whose physical mechanism behind the flux manipulation attributes to evanescent waves instead of surface waves, and is therefore totally different from that of plasmonics.

In this paper, we demonstrate another extraordinary property of the waveguides composed of AENZ materials. Unlike normal waveguides in which the transmission rate strongly depends on the shape of the waveguide boundary, waveguides composed of AENZ materials can achieve high transmission with almost arbitrary shapes of waveguide boundary. Such an amazing property makes AENZ materials perfect wave couplers, with potential applications such as unusual waveguides, wave expanders and compressors, splitters, bends, and devices with combined purposes, etc. Previously transformation optics devices have been proposed to realize similar functions based on the transformation of air, which usually requires different kinds of complex spatial distributions of material parameters^30^,^31^,^32^,^33^,^34^ for different devices. However, here we can use the same homogeneous AENZ media to realize a series of waveguide devices. This property enormously simplifies the design. There is also no requirement on the waveguide area as the waveguide devices based on isotropic ENZ or MNZ materials^12^,^13^,^14^,^15^. By using transformation optics, we can understand the physical origin of such high transmission, and obtain the conditions of total transmission. Finally, we prove our theory by numerical simulations with multilayers consisting of dielectric and negative-permittivity materials.

## Results

**Figure 1:**
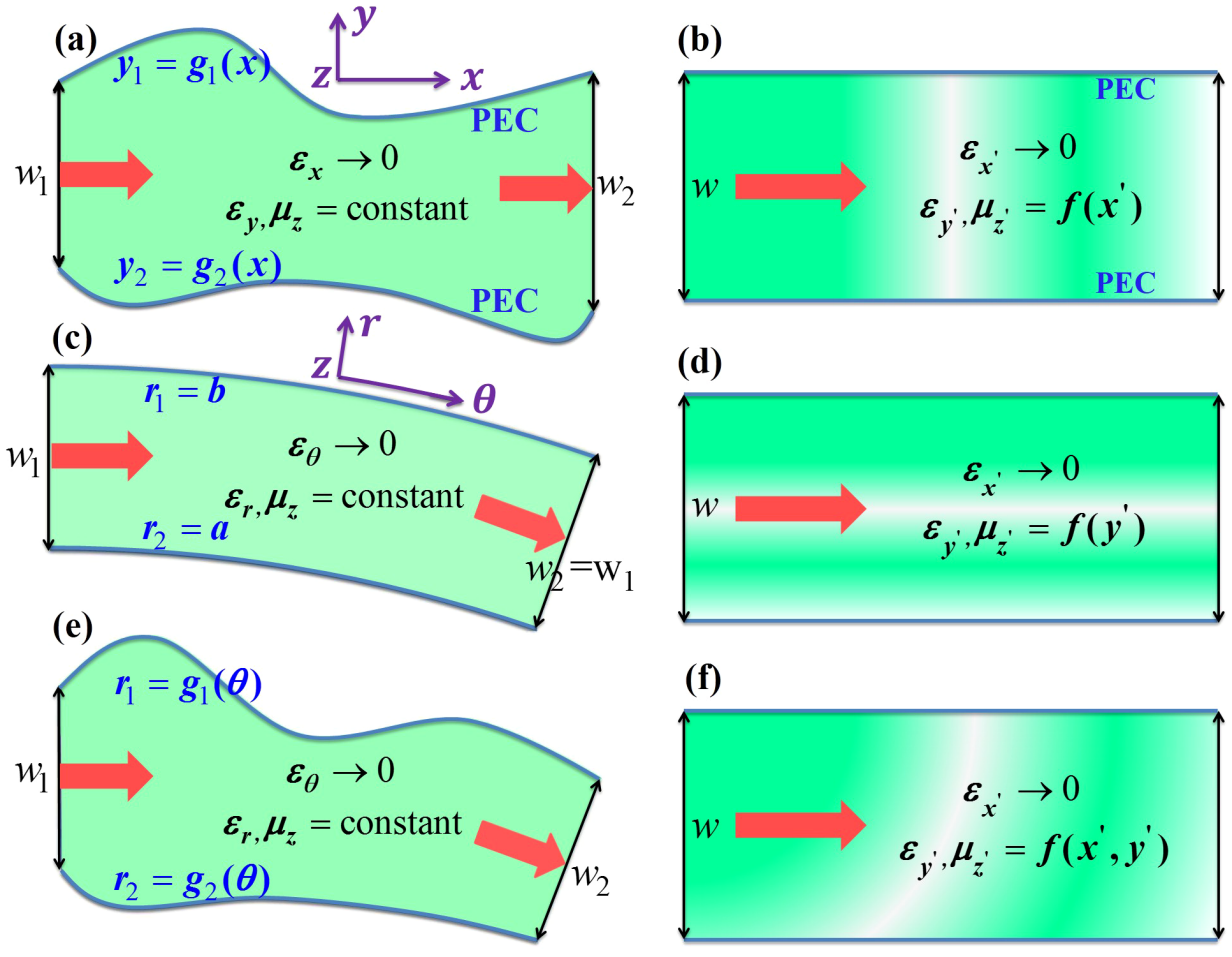
Schematic drawing of the coordinate transformation that express [(a), (c) and (e)] physical waveguides filled with homogeneous AENZ media into [(b), (d) and (f)] virtual straight waveguides filled with inhomogeneous AENZ media.

We consider a waveguide for transverse magnetic (TM) polarized waves with magnetic fields polarized in the *z* direction, constructed with the perfect electric conductor (PEC) boundaries characterized by the functions of *y*_1_ = *g*_1_(*x*) and *y*_2_ = *g*_2_(*x*), respectively, as illustrated in Fig. 1(a). The input and output port surfaces are parallel and have the width of *w*_1_ and *w*_2_, respectively. The waveguide is filled with AENZ media which have a near-zero permittivity in the propagation direction, i.e., *ε_x_* → 0. The other two parameters, i.e. the *y*-component of the relative permittivity *ε_y_* and permeability *μ*, are non-zero and positive constants. To analyze the transmission properties of such a waveguide with irregular boundaries, we apply transformation optics to turn such a waveguide into a straight waveguide having a constant width of *w*, as shown in Fig. 1(b), based on the coordinate mapping of *x*' = *x*, 
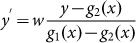
, *z*' = *z*. According to transformation optics, the material parameters of the virtual media are derived as, 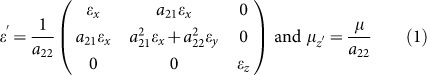
with 
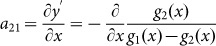
 and 
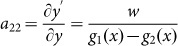
.

Since *ε_x_* → 0, the off-diagonal term of the permittivity tensor *a*_21_*ε_x_* tends to vanish, and the diagonal terms are left with 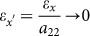 and 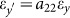. Thus, we can see that the original AENZ media are still AENZ media in the virtual space, but 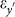 and 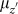 are the functions of *x*'. The wave impedance can be written as, 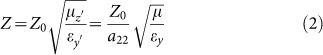
where *Z*_0_ is the impedance of air.

**Figure 2:**
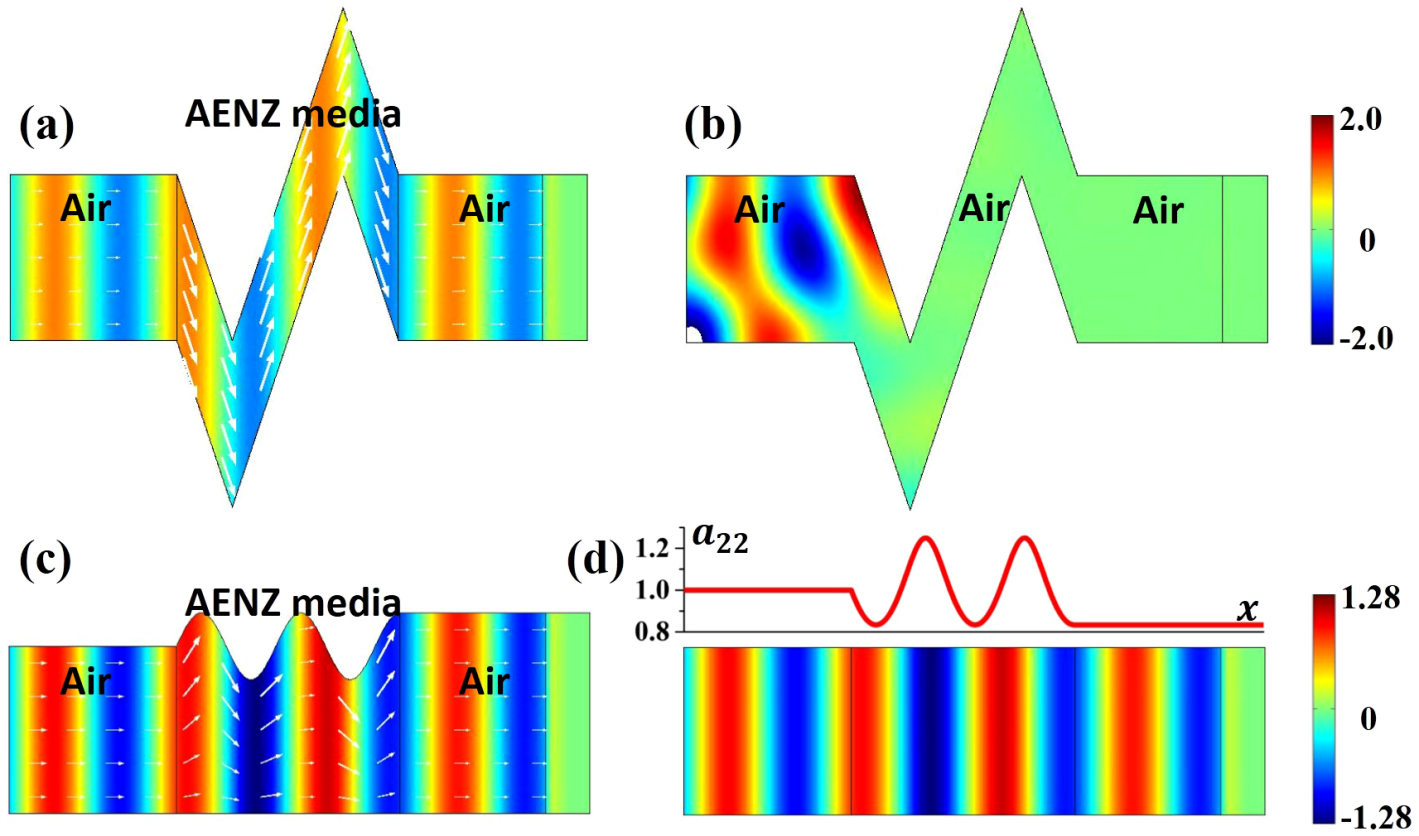
(a) and (b) show the field maps of magnetic field *H_z_* in a waveguide with sharply bent walls when the bending region is filled with AENZ media and air, respectively. (c) and (d) show the field maps of magnetic field *H_z_* in a waveguide expander with a trigonometric boundary and a larger output port, and in the virtual waveguide obtained by transformation optics, respectively. The arrows in (a) and (c) correspond to the Poynting vectors. The upper inset figure in (d) shows the distribution of *a*_22_, which determines the distribution of 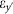 and 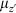.

With Eq. (2), we can evaluate the transmittance of the waves through the waveguide with arbitrary boundary shapes. First, we notice that when *g*_1_(*x*) − *g*_2_(*x*) = *w* is a constant, we have *a*_22_ = 1 and the transformed media exhibits the same impedance as the homogeneous media. Therefore, the transmission would be strictly unity. This is an amazing property. It means that for any waveguides with shapes described by functions of *g*_1_(*x*) or *g*_2_(*x*), as long as *g*_1_(*x*) − *g*_2_(*x*) = *w* is a fixed constant, the transmission rate would always be unity. In Fig. 2(a), we demonstrate a numerical simulation by COMSOL Multi-physics, which shows a TM-polarized wave incident from air into a sharply bent waveguide composed of AENZ materials with *ε_x_* = 0.001 and *ε_y_* = *μ* = 1. The boundaries of the waveguide satisfy *g*_1_(*x*) − *g*_2_(*x*) = *w*. From the distributions of magnetic fields (color-map) and Poynting vectors (arrows) in Fig. 2(a), we clearly observe a perfect transmission of waves through such an unusual waveguide. The almost uniform magnetic field in the *y* direction is a result of the near-zero *ε_x_*. Without the AENZ materials, the dramatic change in the waveguide boundaries would lead to almost total reflection of the incident waves and therefore no transmission, as is shown in Fig. 2(b). We notice that a waveguide cloak has been proposed and experimentally verified by Ma *et al*.^28^, which is based on the same principle. Here, for the first time, we give a rigorous proof by using transformation optics. Our theory not only applies to the cases with fixed *g*_1_(*x*) − *g*_2_(*x*) = *w*, but applies to general cases with arbitrary *g*_1_(*x*) and *g*_2_(*x*), as will be shown below.

Next, we consider another wavy-shaped waveguide whose output port is larger than its input port, as shown in Fig. 2(c). The upper boundary of the waveguide has a trigonometric function, and the AENZ media have the same parameters as those in Fig. 2(a). After transformation to a straight waveguide as shown in Fig. 2(d), 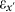 is still taken to be 0.001, while the related parameter *a*_22 _is plotted in the upper figure in Fig. 2(d). Figure 2(c) displays the magnetic fields (color-map) and Poynting vectors (arrows), showing a transmittance larger than 99% even though the impedance is not exactly matched. And the distribution of magnetic fields is in consistent with that inside the straight waveguide shown in Fig. 2(d). The high transmission can be explained by the small impedance variation in the straight waveguide. Since *a*_22_ changes smoothly around unity, the impedance of the system is also varying around the impedance of air. In such cases, high transmission can be generally obtained.

Now, we consider another system illustrated in Fig. 1(c) in which the input and output ports are not parallel, indicating that the AENZ in the waveguide is bent, i.e. *ε_θ_* → 0 instead of *ε_x_* → 0. In our previous work^25^, we have studied this system by transforming it to a straight waveguide with the cylindrical coordinate mapping *x*' = *Rθ*, *y*' = *g*(*r*), *z*' = *z*. We assume that the outer and inner walls are characterized by *r*_1_ = *b* and *r*_2_ = *a*, respectively. The width of the waveguide is fixed at *w*. The parameters of the straight waveguide are, 
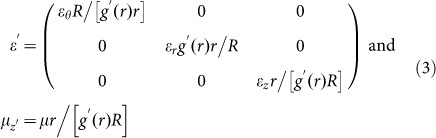
From Eq. (3), it is seen that 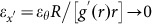 due to *ε_θ_* → 0. However, 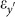 and 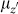 are now functions of *r*, and therefore functions of *y*', as illustrated in Fig. 1(d). Here, we note that this case is different from the case in Fig. 1(b), in which 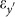 and 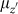 vary in the *x*' direction in the virtual space. In such an inhomogeneous system, evanescent waves are induced, which can efficiently redistribute the energy flow to smoothly match the waveguide modes everywhere^25^,^26^. As a consequence, robust high transmission can be obtained, which can also be understood from the averaging effect on the wave impedance with the formula^29^, 
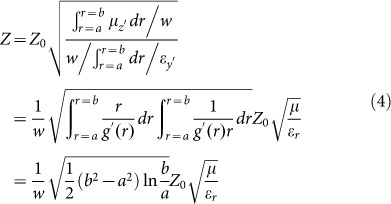
For small variations, the wave impedance is generally close to the impedance of air. For instance, if *b*/*a* = 2 or 20, we will get the ratio of impedance 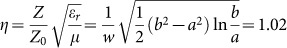 or 1.29. When a film with impedance *Z* placed in the background medium with impedance 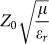, the minimal transmittance can be derived as 
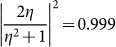
 or 0.938. Thus, it is apparent that the bending waveguides possess robust high transmittance >90% irrespective of the propagation distance^25^,^26^.

If the boundaries are variant, and the input and output port angles of the waveguide are also changed, as shown in Fig. 1(e), we get a combination of the above two cases in Figs. 1(a) and 1(c). Following the above two steps of transformation, such a waveguide filled with homogeneous AENZ media can be transformed into a straight waveguide filled with inhomogeneous AENZ media, whose parameters are functions of both *x*' and *y*' in the virtual space, as illustrated in Fig. 1(f). In the following, we demonstrate some examples of multi-purpose waveguide devices.

**Figure 3:**
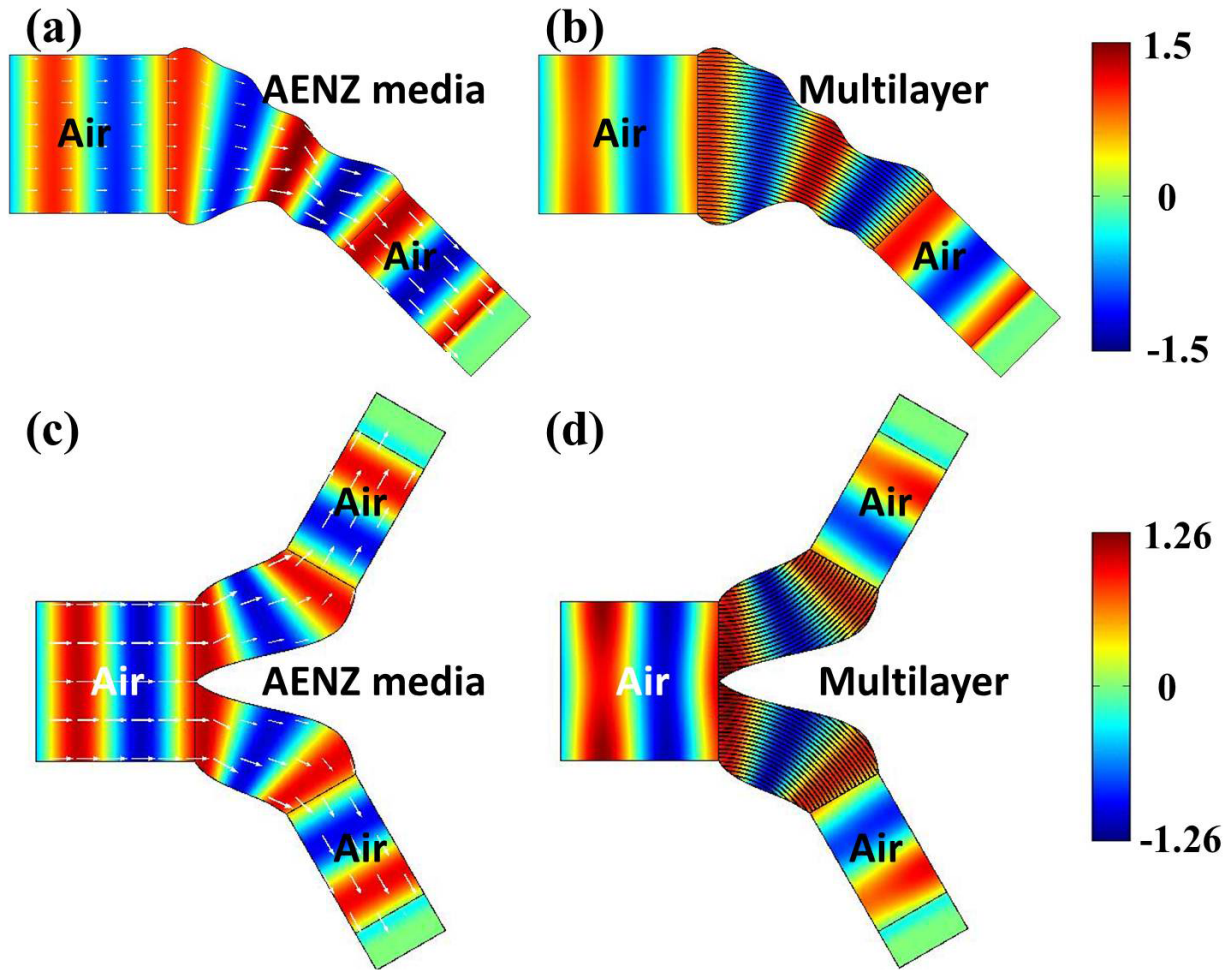
The field map of magnetic field *H_z_* in bending wave [(a) and (b)] compressor and [(c) and (d)] splitter with the bending region filled with [(a) and (c)] lossless AENZ media and [(b) and (d)] lossy multilayers. The multilayers are composed of dielectric and negative-permittivity materials.

The first example is a bending wave compressor in Figs. 3(a) and 3(b). Figure 3(a) presents the distributions of magnetic fields (color-map) and Poynting vectors (arrows) when the relevant parameters of AENZ media are *ε_θ_* = 0.001, *ε_r_* = 1.25 and *μ* = 1. The transmittance is numerically found out to be as high as 0.99. We note that *ε_r_* is chosen to be 1.25, because such a *ε_r_* larger than unity is easier to achieve in practice. It is known that the AENZ media can be readily realized by designing subwavelength multilayers composed of dielectric and negative-permittivity materials^7^,^11^,^35^,^36^,^37^. Suppose that the relative permittivity and filling ratio of the two materials are represented by *ε_d_*, *ε_n_*, *f_d_* and *f_n_*. The effective permittivities parallel and perpendicular to the layers are 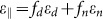 and 
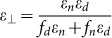
, respectively. If we insist 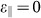 and *ε*_⊥_ = 1, the parameters will have to satisfy 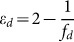 and 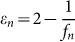. Because of 0 < *f_d_*, *f_n_* < 1, the relative permittivities will satisfy *ε_d_*, *ε_n_* < 1, indicating that one material should have negative permittivity, and the other one should have a relative permittivity less than unity. Here, for simplicity, we choose *ε_r_* = 1.25, which can be realized by the materials with *ε_d_* = 1, *ε_n_* = −5 + 0.5*i*, *f_d_* = 5/6 and *f_n_* = 1/6. *ε_n_* = −5 + 0.5*i* can be realized by air and mesh-based metamaterials in the microwave regime^7^,^38^,^39^. To guarantee the validity of the effective parameters, the lattice constant *a* is chosen as *λ*_0_/30 with *λ*_0_ being the operating wavelength in free space. In Fig. 3(b), we utilize the designed multilayer structure with losses to replace the lossless AENZ media in Fig. 3(a). The magnetic field map shows that the wave front shape of the transmitted wave is the same as that in Fig. 3(a), but the transmittance is reduced a little due to the material losses.

The second example is a bending wave splitter, as shown in Figs. 3(c) and 3(d). Again, the waveguide boundaries are wavy. When the lossless AENZ media with *ε_θ_* = 0.001, *ε_r_* = 1.25 and *μ_z_* = 1 is filled in the bending regions, more than 98% of the incident waves can be transmitted with good wave front shapes, which can be seen from the distributions of magnetic fields (color-map) and Poynting vectors (arrows) in Fig. 3(c). Moreover, such good performance preserves when we use the lossy multilayer to replace the AENZ media, which can be seen from the magnetic field distribution in Fig. 3(d). Due to the existence of material losses, the total transmittance is about 0.75.

## Discussion

In this paper, we propose a new class of waveguide devices based on uniform AENZ media. A big advantage of such waveguide devices is that the high transmission is robust to the shapes of the waveguide boundaries as well as the angle change in the input and output ports. By using transformation optics, we find out the physical origin of such high robust transmission and obtain the condition for total transmission. Such a robust AENZ waveguide with arbitrary shapes has many applications, such as wave expanders, compressors, bends, splitters, and combined purposes. By using a multilayer structure consisting of dielectric and negative-permittivity materials as AENZ media, we numerically demonstrated two examples which match with theory perfectly. Losses do not comprise the robust transmission effect, but only reduce the transmission. In addition, although in the simulations we have set the near-zero permittivity to be 0.001, robust high transmission has also been observed when the near-zero permittivity is 0.01 or 0.1. Comparing with the transformation-optics-based waveguide devices with a complex spatial distribution of parameters, our waveguide devices based on uniform AENZ media could be easier to realize, as we only require the realization of uniform AENZ media.
